# Comparison of intensity modulated radiotherapy plan optimisation methods for a 1.5 T MR‐Linac

**DOI:** 10.1002/acm2.12475

**Published:** 2018-10-29

**Authors:** Robert Chuter, Marcel van Herk, Hafid Akhiat, Peter Voet, Ranald MacKay, Ananya Choudhury, Alan McWilliam

**Affiliations:** ^1^ Christie Medical Physics and Engineering (CMPE) The Christie NHS Foundation Trust Manchester UK; ^2^ Division of Cancer Sciences Faculty of Biology, Medicine and Health University of Manchester Manchester UK; ^3^ NIHR Manchester Biomedical Research Centre Central Manchester University Hospitals NHS Foundation Trust Manchester Academic Health Science Centre Manchester UK; ^4^ Elekta AB Stockholm Sweden; ^5^ Clinical Oncology The Christie NHS Foundation Trust Manchester UK

**Keywords:** MR‐linac, optimisation, treatment planning

## Abstract

**Purpose:**

For the 1.5 T Elekta MR‐Linac it is essential that the optimisation of a treatment plan accounts for the electron return effect on the planned dose distribution. The ability of two algorithms for the first stage fluence optimisation, pencil beam (PB) and Monte Carlo (MC), to produce acceptable plan quality was investigated. Optimisation time for each algorithm was also compared.

**Methods:**

Ten head and neck patients, ten lung patients and five prostate patients were selected from the clinical archive. These were retrospectively planned using a research version of Monaco with both the PB and MC algorithms for the fluence optimisation stage. After full optimisation DVH parameters, optimisation time and the number of Monitor Units (MU) as a measure of plan complexity were extracted.

**Results:**

There were no clinically significant differences between any of the DVH parameters studied or the total number of MUs between using PB or MC for stage 1 optimisation across the three patient groups. However, planning time increased by a factor of ten using MC algorithms for stage 1.

**Conclusion:**

The use of MC calculations compared to PB, for stage 1 fluence optimisation, results in increased planning time without clinical improvement in plan quality or reduction in complexity and is therefore not necessary.

## 1 INTRODUCTION

MR imaging provides excellent soft tissue contrast without the imaging dose associated with CT and Cone Beam CT (CBCT). Improvements in soft tissue contrast are desirable for a number of treatment sites, particularly those where the CBCT imaging may be poor, i.e., pelvis or abdominal tumours.^1^ To enable MR imaging prior to and during treatment, the MR‐Linac^2^ (Elekta, AB, Stockholm, Sweden) from The MR‐Linac Consortium has combined a 1.5 T Philips scanner (Best, The Netherlands) and a 7 MV Elekta linac.

The presence of a 1.5 T magnetic field (B‐field) in the MR‐Linac during beam delivery results in the Lorentz force acting on the secondary electrons, causing them to follow a spiral trajectory. This results in electrons leaving a tissue‐air boundary to be incident again on the exit surface, a process called the Electron Return Effect (ERE)^3^ which can cause a significant increase in the dose to the exit point, for example at the patient's skin or internal air cavities. Which can result in increases of up to 56% for a single beam at highly oblique surfaces.^4^ The B‐field also influences the path of the electrons in tissue with differences seen in the percentage depth dose curves and field profiles.^3^ However, despite these effects, when the planning system accounts for the ERE clinically acceptable plans can be created. This is possible as the Monte Carlo (MC) algorithm accurately models the dose caused by the ERE, which can then be accounted for and removed by the modulation of the fields when IMRT is utilized.^4^, ^5^, ^6^

Planning studies have been conducted for rectum^7^ and lung SBRT^5^ leading to clinically acceptable plans, this has also been shown for pancreatic, head and neck, breast, and lung cases,^6^ where optimising including the B‐field was shown to remove the effects of the ERE.

Plan creation for the MR‐Linac will utilize the Monaco treatment planning system (Elekta AB, Stockholm, Sweden), which uses a two‐stage method to optimize the dose.^8^ The first stage is fluence optimisation and the second is segment shape optimisation. The dose calculation in the second stage is always MC which includes the effect of the 1.5 T B‐field in the dose calculation using the GPUMCD algorithm^9^ and has been shown to produce acceptable results when compared against GEANT4.^10^ However, for the first stage of the optimisation the user has the option to use either a Pencil Beam (PB) or the MC algorithm. The PB algorithm does not account for the B‐field, but has the benefit of being very fast, whereas the MC algorithm will account for the B‐field but is much slower.

It is unknown whether using MC in the first stage (i.e., accounting for the B‐field) improves the final plan quality or if we can use the PB algorithm in stage 1 and recover the plan, accounting for the B‐field, in stage 2 only. The latter option could save a significant amount of time in plan creation. Therefore, it is the purpose of this paper to compare plan quality for plans optimized in stage 1 with PB with those optimized with the MC algorithm. Additionally, a comparison of the MUs required for each plan as a surrogate for plan complexity^11^, ^12^ and the total time taken to optimize using each method will be compared.

## 2 MATERIALS AND METHODS

### 2.A Patient selection

Ten head and neck, ten lung and five prostate patients, all treated curatively at the authors institution, were randomly selected from the clinical archive. All target volumes and organs at risk (OAR) had been delineated at the time of planning by a radiation oncologist specialising in the relevant treatment site.

### 2.B Choice of segmentation parameters

To investigate whether the choice of segmentation parameters could significantly affect the results, a range of segmentation parameters were investigated for a representative lung plan. A lung plan was chosen as this treatment site would, potentially, show a larger effect from the ERE due to the greater number of air‐tissue interfaces. The following parameters were varied: minimum segment area, minimum segment width, minimum MU per segment and maximum number of segments per plan. Three combinations were tested, aiming to cover the range of likely optimisation parameters used clinically. One allowed small segments and 180 segments per plan, one only allowed large segments and only 60 segments per plan, whilst the final test had intermediate parameters (Table 1).

**Table 1:** For a representative lung patient showing the segment parameters used to test the sensitivity to these for the results obtained. The parameters in bold are the ones selected for plan optimisation of the lung plans

Segment test	Complex	Intermediate	Simple
Minimum segment area (cm^2^)	3.0	**9.0**	12.0
Minimum segment width (cm)	0.5	**0.5**	1.0
Minimum MU/segments	3.0	**3.0**	4.0
Maximum # of seg per plan	180	**90**	60

### 2.C Creation of treatment plans

For plan optimisation a research version of Monaco was used in conjunction with an MR‐Linac specific beam model. For the head and neck (H&N) plans Monaco v5.19.00 was used and for the lung and prostate plans Monaco v5.19.02 was utilized, all optimized as 7‐field step and shoot IMRT. Head and neck plans were prescribed to 6600 cGy in 30 fractions, lung plans to 5500 cGy in 20 fractions and the prostate plans to 6000 cGy in 20 fractions. Final dose calculations were performed with a 1% statistical uncertainty with the GPUMCD algorithm and accounting for the 1.5 T B‐field.^9^ Plans were optimized to meet the standard clinical departmental constraints for target coverage and OAR doses.

For each patient, the first plan was fully optimized using the PB algorithm for the fluence optimisation (stage 1). The second plan was optimized using the same objective functions and the GPUMCD algorithm for the fluence optimisation stage. Both optimisation arms performed a GPUMCD optimisation as standard in stage 2. This process was automated utilising Elekta's research automation toolkit which is an API allowing the software to communicate with the Monaco user interface. All calculation and segmentation parameters were kept constant between plans; details of these are shown in Table 2. This ensures that the plan comparison is between the stage 1 dose calculation and is not influenced by the objective functions or optimisation parameters.

**Table 2:** Calculation and segmentation parameters for the three different tumour sites

	H&N	Lung	Prostate
Beam model	ElektaMRLv509	ElektaMRLv519	ElektaMRLv519
Energy	8 MV FFF	7 MV FFF	7 MV FFF
Grid spacing (cm)	0.3	0.3	0.3
Statistical uncertainty Stage 1 (%)	3	3	3
Statistical uncertainty Stage 2 (%)	1	1	1
Minimum segment area (cm^2^)	2.0	9.0	5.0
Minimum segment width (cm)	0.5	0.5	1.0
Minimum MU/segment	4.0	3.0	4.0
Maximum # segments per plan	90	90	80

### 2.D Analysis of results

The relevant dose statistics to the PTVs and the OARs important for each site were recorded, as documented in Table 3. For each of the sites the maximum dose to 2 cc of the skin, defined here as a 5 mm contraction from the external contour, was extracted. Due to the potential increase in dose at the lung‐tissue interface the maximum 2 cc dose to the lung surface was extracted, defined as a 5 mm thick layer around the inside of the lung contour. A planning risk volume (PRV) was used around the spinal cord and brainstem, where applicable, expanding isotropically by 5 mm. A copy of the PTV was created which excluded any volume outside the skin contracted by 5 mm, to remove the influence of the build‐up region near the patient's skin (PTV IMRT).

**Table 3:** DVH parameters and structures extracted for each of the treatment sites investigated

H&N		Lungs		Prostate	
PTV IMRT	D95%	PTV IMRT	D98%	PTV IMRT	D95%
Spinal Cord PRV	1 cc (Max)	PTV IMRT	D2%	PTV	2 cc (min)
Brainstem PRV	1 cc (Max)	Lung	Mean	PTV	2 cc (max)
Lt/Rt Parotid	Mean dose	Lung	V5	Rectum	2 cc (min)
Larynx	Mean dose	Lung	V10	Rectum	2 cc (max)
Skin	Max 2 cc	Lung	V20	Rectum	Mean
		Lung	V30	Rectum	V30
		Heart	V10	Rectum	V40
		Heart	V20	Skin	Max 2 cc
		Heart	V30		
		Heart	V40		
		Lung Surface	Max 2 cc		
		Skin	Max 2 cc		

The magnitude of the change in dose to the target and OARs between the two algorithms was compared. The results are presented as box and whisker plots with the boxes marking the 5^th^ and 95^th^ percentiles, the band marks the median, the whiskers mark the maximum and minimum values and the stars indicate the mean value. A paired t‐test was performed between the PB and MC dosimetric statistics to highlight any statistically significant differences.

The time taken to optimize each plan was measured, from initiating stage 1 optimisation to the end of the dose calculation, to assess the usability of each method. Additionally, the total number of MUs was also recorded to provide an estimate of deliverability.^11^

## 3 RESULTS

Different segmentation parameters were tested for a representative lung plan. Table 4 shows that the number of MUs was much greater for plans that allowed more segments but the MUs were not significantly (*P* < 0.01) different between PB and MC for any choice of segmentation parameters. Additionally, regardless of choice of segmentation parameters the time taken using the MC algorithm was always much larger but decreases from being 20 times larger to 6 times larger going from complex to more simple segments. The variation in the DVH parameters for the target and OARs can be seen in Table 5. The differences between using the PB and MC algorithms for the three different segmentation parameters are all below 2% and mostly below 1%, highlighting that segmentation parameters have a small effect on the outcome of this comparison.

**Table 4:** Time and MU results for three segmentation parameter choices used for a Lung plan. The intermediate segment parameters were utilized for the rest of the patients, these are highlighted in bold

Segmentation test	Complex	Intermediate	Simple
MU			
PB‐MC	1320.1	**1110.2**	869.5
MC‐MC	1530.7	**1138.6**	889.05
Optimisation time (mins)			
PB‐MC	20.08	**17.35**	14.14
MC‐MC	149.86	**192.71**	81.21

**Table 5:** DVH parameters for the PTV IMRTs, both lungs, heart, skin and lung surface for the three different segmentation parameter choices used (see Table 1) for a representative lung plan. Differences above 1% are highlighted in red/italics. The intermediate segment parameters were utilized for the rest of the patients, these are highlighted in bold

	Complex			Intermediate			Simple		
	PB‐MC	MC‐MC	Diff (%)	PB‐MC	MC‐MC	Diff (%)	PB‐MC	MC‐MC	Diff (%)
PTV IMRT 98% (cGy)	5359.8	5388.4	*−*0.8	**5320.3**	**5365.2**	*−*0.5	5267.7	5337.8	***−1.3***
PTV IMRT 2% (cGy)	5636.2	5614.2	0.5	**5647.3**	**5621.2**	0.4	5666.4	5633.4	0.6
Lungs V5 Gy (%)	58.7	58.9	*−*0.4	**58.8**	**59.2**	*−*0.2	59.9	59.2	0.7
Lungs V10 Gy (%)	51.2	51.7	*−*0.7	**51.2**	**51.9**	*−*0.5	52.3	52.0	0.4
Lungs V20 Gy (%)	25.6	26.4	*−*0.8	**25.7**	**26.5**	*−*0.8	26.5	26.5	0.1
Lungs V30 Gy (%)	9.5	9.7	*−*0.2	**9.5**	**9.7**	*−*0.3	9.6	9.6	0.0
Mean dose to both lungs (cGy)	1327.1	1347.6	***−1.8***	**1324.3**	**1348.4**	***−1.5***	1349.6	1347.7	0.1
Heart V10 Gy (%)	32.9	33.4	*−*0.5	**33.2**	**33.7**	*−*0.5	34.0	33.8	0.2
Heart V20 Gy (%)	24.8	25.6	*−*0.8	**25.1**	**25.9**	*−*0.8	25.9	26.5	−0.6
Heart V30 Gy (%)	18.0	18.5	*−*0.4	**17.9**	**18.3**	*−*0.5	18.2	19.0	−0.8
Heart V40 Gy (%)	12.4	12.6	*−*0.1	**12.4**	**12.5**	*−*0.2	12.8	13.0	−0.1
Skin 2 cc (max) (cGy)	2653.4	2651.3	***1.2***	**2619.7**	**2588.0**	0.1	2581.2	2547.5	***1.3***
Lung Surface 2 cc (max) (cGy)	5635.6	5611.6	0.5	**5645.8**	**5615.5**	0.4	5660.0	5630.8	0.5

The average MUs and optimisation time over the number of patients planned for each treatment group are shown in Table 6. This shows the average time taken to optimize using the PB and MC algorithms, as well as the average number of MUs. The standard deviation is shown in parentheses.

**Table 6:** The mean and standard deviation in the number of MUs and time taken to optimise the plans is shown. The two different algorithms used for the fluence optimisation stage, PB and MC are shown for three different anatomical sites. The *P*‐values for a paired t‐test are also shown in bold

Mean (SD)	Patient group		
	H&N	Lung	Prostate
Monitor units (MU)			
PB	1494.6 (152.9)	1377.4 (328.5)	1508.8 (105.3)
MC	1517.9 (169.9)	1410.4 (328.4)	1553.7 (173.2)
* P*‐value	**0.63**	**0.17**	**0.24**
Optimisation time (mins)			
PB	38.9 (15.8)	24.9 (7.3)	29.7 (2.5)
MC	480.4 (176.9)	296.7 (136.3)	263.2 (77.9)
* P*‐value	**≪0.001**	**0.001**	**0.002**

Table 6 shows that the number of MUs delivered with plans optimized with PB and MC for stage 1 are not significantly different for any of the treatment sites investigated. This indicates that the plans would take no longer to deliver or are no more complex in either arm. However, the difference in times it takes to optimize the plans is highly significant. It takes approximately ten times longer to optimize a plan using MC for stage 1 than it does with PB for stage 1. For head and neck plans the differences increase to over 12 times longer but for prostate plans this is 9 times longer.

**Figure 1:**
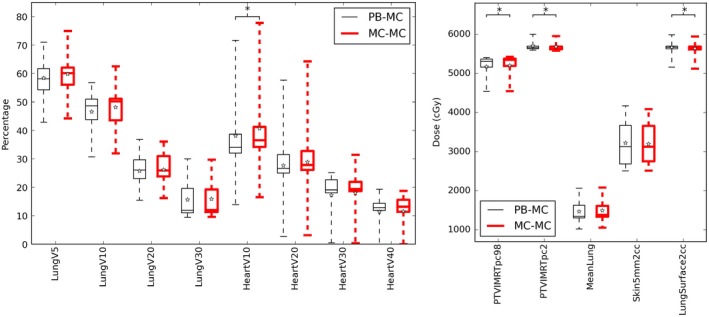
DVH parameters for ten lung patients optimized with a PB algorithm (thin black lines) and MC algorithm (thick red lines) for fluence optimisation. The boxes mark the 5^th^ and 95^th^ percentiles, the band marks the median, stars mark the mean and the whiskers mark the maximum and minimum values. A * indicates a significance of *P *<* *0.01.

**Figure 2:**
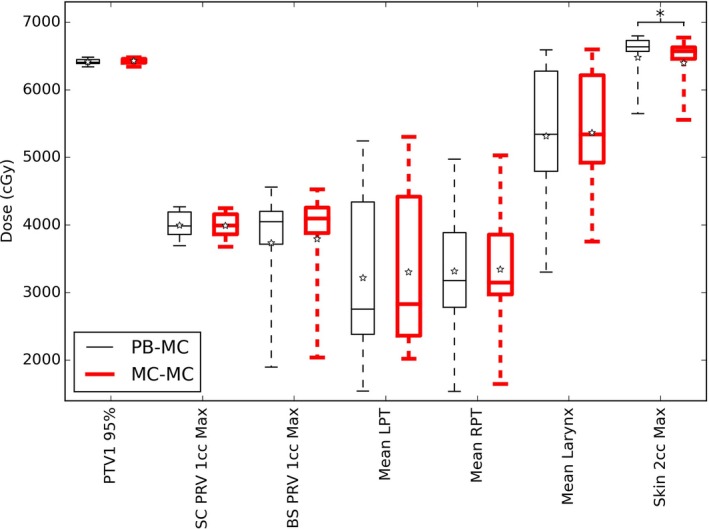
DVH parameters for ten H&N patients optimized with a PB algorithm (thin black lines) and MC algorithm (thick red lines) for fluence optimisation. The boxes mark the 5^th^ and 95^th^ percentiles, the band marks the median, stars mark the mean and the whiskers mark the maximum and minimum values. A * indicates a significance of *P *<* *0.01.

**Figure 3:**
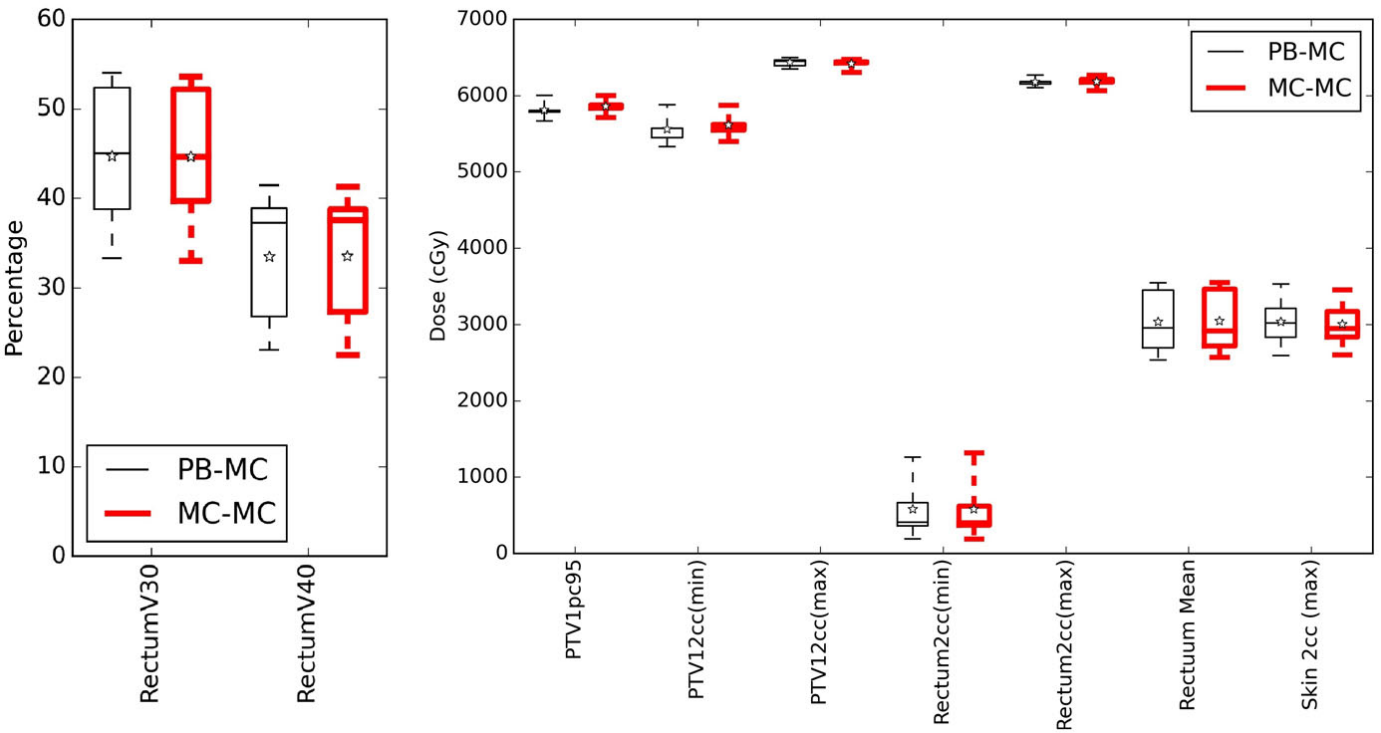
DVH parameters for five prostate patients optimized with a PB algorithm (thin black lines) and MC algorithm (thick red lines) for fluence optimisation. The boxes mark the 5^th^ and 95^th^ percentiles, the band marks the median, stars mark the mean and the whiskers mark the maximum and minimum values.

The DVH parameters evaluated (Table 3) for lung patients with the PB and MC algorithm are shown in Fig. 1. Figures 2 and 3 show the same data for ten head and neck patients, and five prostate patients respectively.

Most parameters showed with little difference between the two optimisation arms. The DVH parameters that showed statistically significant difference (at *P* < 0.01 which was chosen due to the low number of patients) are denoted in the plots with a *. The H&N patients show a significant difference for the maximum dose to 2 cc of the skin, with the PB plans being 1.3% higher which is not considered clinically significant. The prostate plans showed no significant differences between the optimisation arms.

**Figure 4:**
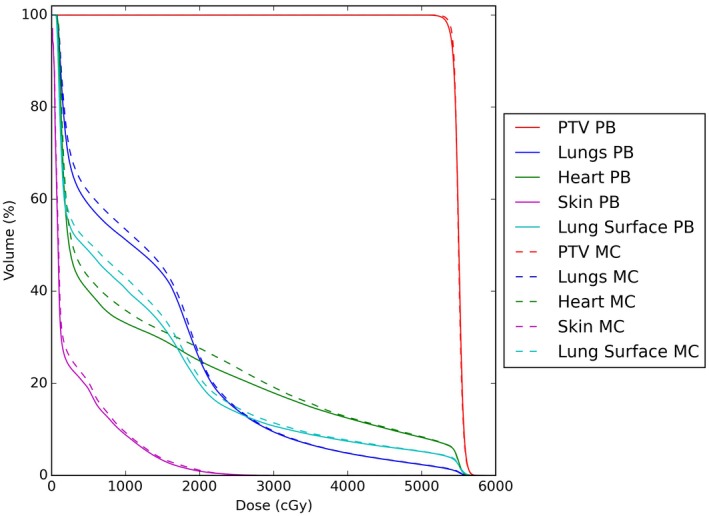
DVHs for a representative lung patient optimized with a PB algorithm (thick lines) and MC algorithm (dashed lines) for fluence optimisation (stage 1).

Some parameters were statistically significant for the lung cases but these were not considered clinically significant on discussion with a consultant oncologist. Specifically, a difference for the Heart V10 was seen of 2.4%, with the MC arm showing a higher dose. Figure 4 shows the DVHs for a representative lung patient optimized with a PB algorithm (thick lines) and MC algorithm (dashed lines) for fluence optimisation.

## 4 DISCUSSION

This work has investigated the effect of using PB or MC algorithms for fluence optimisation in creating plans with Monaco for the MR‐Linac for multiple sites. The presence of the B‐field results in the ERE, that is only reproduced with the MC dose calculation. However, using a MC dose calculation will take a significantly longer time in an optimisation process compared to PB. This work has demonstrated that there were no clinically significant changes in the DVH parameters between using the two algorithms for the first stage of the optimisation. Nonetheless there is a large increase (~10 times) in the time taken to optimize when using the MC algorithm. Only five parameters showed a statistically significant dosimetric difference between plans produced using the MC or PB algorithms. These differences between the arms were all less than 2.5% and on discussion with a consultant oncologist were not considered clinically important.

This work has investigated two optimisation arms, PB or MC, for head and neck plans, lung plans and prostate plans to cover a range of anatomical sites with varying amounts of inhomogeneities present. Interestingly, even the lung plans showed minimal differences between the optimisation arms. Because of the large inhomogeneities and tissue/air interfaces it was expected that using the MC algorithm for fluence optimisation would work better as it accounts for the B‐field and associated ERE during optimisation. There were statistically significant differences for four of the DVH parameters for the lung plans but none of these were considered clinically significant. Apparently the changes in dose distribution due to ERE are so localized that the segment weight optimization in the second stage, where MC accounting for the B‐field was used, could fully recover the plan quality.

Several papers have shown that Monaco is capable of producing clinically acceptable plans for linacs with a 1.5 T B‐field.^5^, ^6^, ^7^ However, to our knowledge none have investigated the effect of the fluence optimisation method on plan quality. This is the first paper to investigate whether the choice of algorithm in the first stage of optimisation makes a difference on plan quality for the MR‐Linac. The results obtained here could also be true at 0 T, applicable for standard linacs but this has not been investigated here.

One of the limitations of this study was that the segmentation parameters were kept constant for each plan site. We initially tested several sets of parameters for a lung plan. Changing from 60 to 180 segments per plan, as well as from 3 cm^3^ to 12 cm^3^ for the minimum segment area kept differences between PB and MC algorithms below 1% for most DVH parameters, with only 5 parameters between 1% and 2%. Therefore, we believe that the choice of segmentation parameters does not change the overall conclusion. We have investigated head and neck, thorax and pelvis regions to have a range of anatomy, and since in all three the first stage optimization made no difference we expect this conclusion to hold for further treatment sites.

The number of MUs has been used as a surrogate measure for complexity and whilst other metrics have been proposed,^12^ the number of MUs is still thought to give an indication of the complexity of a plan^11^, ^12^ and is easily extracted from the plans. The optimisation method showed no significant difference in the plan MU, indicating no measurable difference in complexity between plans.

This work illustrates that using the faster PB algorithm for fluence optimisation does not degrade the plan quality or reduce plan deliverability.

## 5 CONCLUSION

The MR‐Linac will utilize the Monaco TPS which uses two stages for plan optimisation, stage 1 — fluence optimisation and stage 2 — segment optimisation. This investigation has shown that due to increased planning time without significant improvement in plan quality, the use of MC for the fluence optimisation stage is not necessary.

## CONFLICT OF INTEREST

The Christie is a member of the Elekta MR‐Linac consortium from which we have received financial and technical support under a research agreement with Elekta AB. However, Elekta had no part in the design or execution of the study.

## References

[1] Lagendijk JJ , Raaymakers BW , van dBC , Moerland MA , Philippens ME , van Vulpen M . MR guidance in radiotherapy. Phys Med Biol 2014;59:R349–R369. 25322150 10.1088/0031-9155/59/21/R349

[2] Lagendijk JJ , Raaymakers BW , Raaijmakers AJ , et al. MRI/Linac integration. Radiother Oncol. 2008;86:25–29. 18023488 10.1016/j.radonc.2007.10.034

[3] Raaijmakers AJ , Raaymakers BW , Lagendijk JJ . Integrating a MRI scanner with a 6 MV radiotherapy accelerator: dose increase at tissue‐air interfaces in a lateral magnetic field due to returning electrons. Phys Med Biol. 2005;50:1363–1376. 15798329 10.1088/0031-9155/50/7/002

[4] Raaijmakers AJ , Raaymakers BW , van der Meer S , Lagendijk JJ . Integrating a MRI scanner 537 with a 6 MV radiotherapy accelerator: impact of the surface orientation on the entrance and 538 exit dose due to the transverse magnetic field. Phys Med Biol. 2007;52:929–939. 17264362 10.1088/0031-9155/52/4/005

[5] Menten MJ , Fast MF , Nill S , Kamerling CP , McDonald F , Oelfke U . Lung stereotactic body radiotherapy with an MR‐linac – quantifying the impact of the magnetic field and real‐time tumor tracking. Radiother Oncol. 2016;119:461–466. 27165615 10.1016/j.radonc.2016.04.019 PMC4936791

[6] Chen X , Prior P , Chen G‐P , Schultz CJ , Li XA . Technical Note: dose effects of 1.5T transverse magnetic field on tissue interfaces in MRI‐guided radiotherapy. Med Phys. 2016; 43: 4797–4802. 27487897 10.1118/1.4959534

[7] Uilkema S , van der Heide U , Sonke J‐J , Moreau M , van TB , Nijkamp J . A 1.5 T transverse magnetic field in radiotherapy of rectal cancer: impact on the dose distribution. Med Phys 2015;42:7182–7189. 26632072 10.1118/1.4936097

[8] Semenenko V , Reitz B , Day E , Sharon Qi X , Miften M , Allen LX . Evaluation of a commercial biologically based IMRT treatment planning system. Med Phys. 2008;35:5851–5860. 19175141 10.1118/1.3013556

[9] Hissoiny S , Raaijmakers AJ , Ozell B , Despres P , Raaymakers BW . Fast dose calculation in magnetic fields with GPUMCD *Phys* . Med. Biol. 2011;56:5119–5129. 10.1088/0031-9155/56/16/003 21775790

[10] Ahmad SB , Sarfehnia A , Paudel MR , et al. Evaluation of a commercial MRI Linac based Monte Carlo dose algorithm with GEANT4. Med Phys. 2016;43:894–907. 26843250 10.1118/1.4939808

[11] Craft D , Süss P , Bortfeld T . The tradeoff between treatment plan quality and required number of monitor units in intensity modulated radiotherapy. Int J Radiat Oncol Biol Phys. 2007;67:1596–1605. 17394954 10.1016/j.ijrobp.2006.11.034

[12] McNiven A , Sharpe M , Purdie T . A new metric for assessing IMRT modulation complexity and plan deliverability *Med* . Phys. 2010;37:505–515. 10.1118/1.3276775 20229859

